# Post-synthetic modification of imine linkages of a covalent organic framework for its catalysis application

**DOI:** 10.1039/d0ra02142c

**Published:** 2020-05-05

**Authors:** Qianqian Yan, Huanjun Xu, Xuechao Jing, Hui Hu, Shenglin Wang, Chaoyuan Zeng, Yanan Gao

**Affiliations:** Key Laboratory of Ministry of Education for Advanced Materials in Tropical Island Resources, Hainan University No. 58, Renmin Avenue Haikou 570228 China ygao@hainanu.edu.cn; School of Science, Qiongtai Normal University China; Liaocheng Luxi Polycarbonate Co. Ltd Liaocheng 252000 China

## Abstract

Post-synthetic modification has been the most powerful strategy for covalent organic frameworks (COFs) for their functionalization in many fields. This strategy is typically achieved through the quantitative reaction between existing reactive sites on the linkers (building units) and incoming functional groups. However, usage of linkages (bonds formed to construct COFs) for the post-synthetic modification still remains limited. Herein, we develop a new post-synthetic modification route that is based on the modification of linkages. With this strategy, the imine linkages of a two-dimensional (2D) COF, TFPPy–PyTTA–COF, have been transformed into amine linkages to give the amine-linked isostructure with retention of crystallinity and porosity. The subsequent aminolysis of the amine linkages with 1,3-propane sultone and further metathetical reaction with cobalt acetate [Co(OAc)_2_] enable the introduction of cobalt alkyl sulfonate to the one-dimensional (1D) channel walls of the COF. The resulting ionic COF with coupled Co^2+^ in the frameworks shows excellent catalytic activity and good recyclability towards the cycloaddition reactions of epoxides and CO_2_. This strategy is of interest as it opens a way to use linkage modification for exploring the potential of COFs for different applications.

## Abstract 2

A new approach for post-synthetic modification of covalent organic frameworks has been developed based on the modification of the linkages and the resulting COF exhibited excellent catalytic performance towards cycloaddition of epoxides and CO_2_.

## Graphical abstract

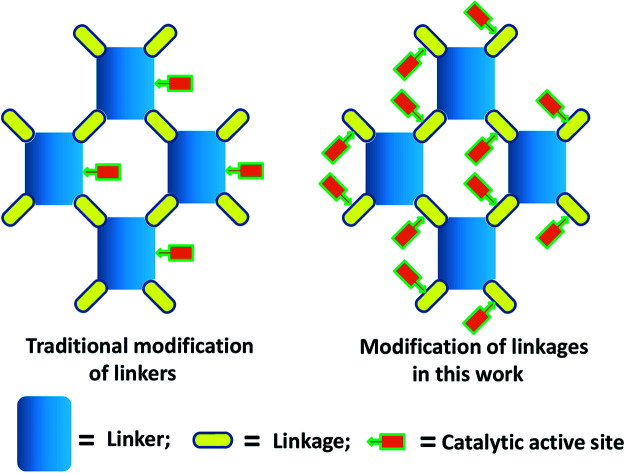

## 1. Introduction

Covalent organic frameworks (COFs) are a fascinating class of crystalline porous polymers that enables the precise integration of building units into periodically ordered polygonal skeletons and aligned built-in pores.^1,2^ They are formed from organic building units (as linkers) linked by covalent bonds (as linkages) based on reticular chemistry. Owing to their special structures and properties, COFs have shown great potential in areas such as gas adsorption,^3,4^ separation,^5,6^ proton conduction,^7,8^ energy storage^9,10^ and optoelectronics.^11–13^ Especially in the catalysis field, the flexible regulation of pore size, shape, and size distribution, large surface area, as well as easy introduction of catalytic sites to the skeletons of COFs make them promising materials for catalysis applications.^14^ Targeted spatial arrangement and controlled density of catalytic active sites in the framework endow COFs with cooperative catalysis character and high catalytic activity. Most 2D COFs adopt an AA stacking model that presents 1D channel structure,^15^ and 1D channel structure can also be found in all 3D COFs if observe from a certain facet. The 1D channel is open and accessible with only two windows on both ends, which allows fast diffusion of reagent, substrate and product through the framework. Besides, akin to other heterogeneous catalysts, the ability to separate for recycling is much attractive in large-scale reactions, especially for costly separation and waste disposal. These advantages make it capable to catalyze a variety of useful organic reactions including Suzuki–Miyaura coupling,^16^ Heck reaction,^17^ Michael addition reaction,^18^ Diels–Alder reaction,^19^ Henry reaction,^20^ epoxidation of alkenes,^21^ Heck–epoxidation tandem reaction,^22^ oxidation–Knoevenagel cascade reaction,^23^ and addition–oxidation cascade reaction.^24^

Post-synthetic integration of catalytic active sites into a COF skeleton is, by far, the most powerful strategy to construct catalytic COFs. In contract to bottom-up strategy, this approach can reduce the impact of bulky catalytic sites on COF crystallinity, and the undesired effect of harsh solvothermal conditions on the catalytic sites. With this strategy, the pristine COFs were first assembled *via* building units that possess reactive active groups such as organic ligand, hydroxyl, azide, or alkyne groups.^25^ Post-synthetic modification was achieved through subsequent quantitative addition or click reaction with these groups to anchor desired catalytic species, generating new pore wall surfaces with uniformly distributed catalytic sites across the frameworks. A variety of catalytic active species, like metal ions, metal nanoparticles, chiral moieties and ionic liquids have been introduced to obtain catalytic COF materials that exhibited excellent catalytic performance in various organic reactions.^26^

By far, almost all post-synthetic functionalization has been achieved through the modification of linkers. However, direct modification of the linkages to introduce functional groups has been rarely reported.^27^ It will be of great importance to develop novel routes that use the linkages for functional modification which can not only avoid the incompatibility of functional groups with COF synthesis conditions or intrinsic reversibility of linkages but also expand the number and type of reactive sites to sequentially anchor more functional groups to enrich the performance of COFs for multifunctional applications.

**Scheme 1:**
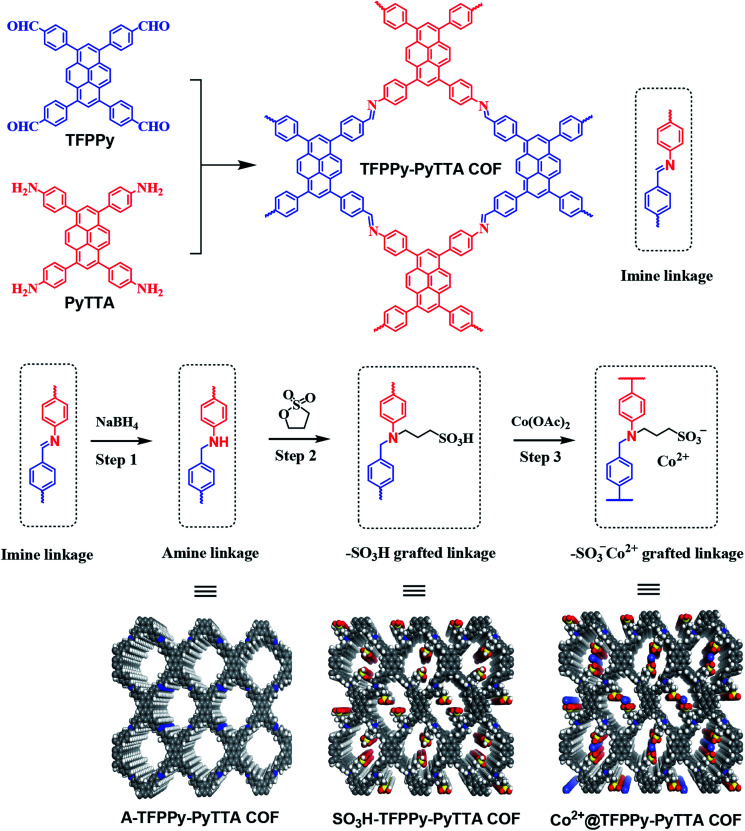
Synthesis procedure of Co^2+^@TFPPy–PyTTA–COF.

Here, we introduce an approach to modify postsynthetically the pore wall surfaces through a three-step modification step to introduce functionalities onto the linkages and tailor the pores (Scheme 1). Specifically, a layered imine-linked COFs, TFPPy–PyTTA–COF^28,29^ was used as starting material and first subjected to reductive conditions to convert the imine linkages quantitatively and present the corresponding amine-linked COF, A-TFPPy–PyTTA–COF, without loss of their underlying topology, crystallinity, and permanent porosity. In second step, the amine linkages undergo aminolysis reaction with 1,3-propane sultone, giving a sulfonic acid functionalized COF, SO_3_H@TFPPy–PyTTA–COF. Considering the functional diversity of ionic architectures, SO_3_H@TFPPy–PyTTA–COF was further modified through metathetical reaction of alkyl sulfonic acid and cobalt acetate, leading to a new ionic framework, Co^2+^@TFPPy–PyTTA–COF. In this context, the catalytic performance of the resulting Co^2+^@TFPPy–PyTTA–COF was evaluated by the cycloaddition of epoxides and CO_2_. Our experimental results show that Co^2+^@TFPPy–PyTTA–COF exhibited excellent catalytic activity towards the cycloaddition reaction with good recyclability.

## 2. Experimental

### 2.1. Materials

All starting materials and solvents, unless otherwise specified, were obtained from commercial resources and used without further purification. Two building units, 1,3,6,8-tetrakis(4-formylphenyl)pyrene (TFPPy) and 4,4′,4′′,4′′′-(pyrene-1,3,6,8-tetrayl)tetraaniline (PyTTA) were synthesized according to published procedures.^30,31^ All reactions were performed at ambient laboratory conditions, and no precautions taken to exclude oxygen or atmospheric moisture unless specified.

### 2.2. Synthesis

#### Synthesis of TFPPy–PyTTA–COF

TFPPy (62.0 mg, 0.1 mmol), and PyTTA (56.8 mg, 0.1 mmol) were placed in a glass ampule vessel (10 mL), followed by adding a solution of mesitylene/1,4-dioxane/*N*,*N*-dimethylacetamide (10/10/7 by volume; 2.7 mL). The mixture was sonicated for 10 min and 0.1 mL of 3.0 M acetic acid was rapidly added. The vessel was then flash frozen in liquid nitrogen and degassed by three freeze–pump–thaw cycles. The system was sealed with a flame, and then heated at 140 °C for 3 days. The resulting precipitate are filtered out, washed thoroughly with DMF and chloroform to give a yellow colored powder, which was dried at 120 °C under vacuum overnight to give the desired product in 78% yield.

#### Synthesis of Co^2+^@TFPPy–PyTTA–COF

TFPPy–PyTTA–COF (30 mg) was added into 20 mL of 1.0 M NaBH_4_ alcohol solution, and the mixture was stirred for 30 min at room temperature. The sample was separated by filtration and washed with alcohol. The freshly made sample *i.e.* A-TFPPy–PyTTA–COF, was then transferred into a one-neck round flask charged with 40 mg of 1,3-propane sultone in 10 mL anhydrous toluene. The mixture was heated at 120 °C for 2 h. After cooling the mixture to room temperature, the precipitate of product was collected by filtration, washed with acetone thoroughly, giving a corresponding sulfonic acid functionalized product of SO_3_H@TFPPy–PyTTA–COF (87% yield). After that, the as-synthesized SO_3_H@TFPPy–PyTTA–COF was added into 37.5 mg of cobalt acetate tetrahydrate [Co(OAc)_2_·4H_2_O] in 10 mL of anhydrous alcohol. The mixture was stirred at room temperature for 12 h, and the precipitate of product was separated by filtration, thoroughly washed with alcohol and dried at 80 °C under vacuum overnight to get the targeted product of Co^2+^@TFPPy–PyTTA–COF in 92% yield.

### 2.3. General procedures for cycloaddition of epoxides with CO_2_

All the reactions were carried out in a sealed Teflon-lined autoclave (50 mL). The mixture of epoxides (7.5 mmol) and Co^2+^@TFPPy–PyTTA–COF (24.4 mg) were taken into the reactor without solvent. The air in the reactor was removed by CO_2_ purge. The reactor was pressurized with CO_2_ (3.0 MPa), and then the temperature was raised to 100 °C. The reaction was conducted for 24 h. After reaction, the reactor was cooled down in ice cold water. Unreacted CO_2_ was slowly vented out, and Co^2+^@TFPPy–PyTTA–COF was separated by centrifugation. The products were analyzed by gas chromatography (Agilent HP 6890 A) equipped with a capillary column (HP-5, 30 m × 0.25 mm) and a flame ionization detector. The yield of products was determined by using toluene as internal standard.

### 2.4. Characterization

Fourier transform infrared (FT-IR) spectra were recorded using KBr pellets on a Bruker model TENSOR 27 spectrophotometer. Power X-ray diffraction (PXRD) measurements were recorded on a PANalytical X'Pert model Pro Multipurpose Diffractometer using Cu K_α_ radiation at 40 kV and 40 mA. The signals were collected from 2*θ* of 2.5–30° at 0.03° step scan with exposure time of 10 s per step. Low-pressure (0–110 kPa) N_2_ gas sorption isotherms were measured volumetrically at 77 K using a Quantachrome Autosorb-iQ2 analyzer with ultrahigh-purity gases. The fresh samples were activated at 100 °C for 15 h under high vacuum prior to analysis. Brunauer–Emmett–Teller (BET) model was used to determine the specific surface areas using desorption branches over *P*/*P*_0_ of 0.01–0.05. In all isotherm plots, closed circles describe adsorption data points and open circles are used to represent desorption data points. The pore size distribution was evaluated by the nonlocal density functional theory (NLDFT) method. Thermogravimetric analysis (TGA, STA449F3, NETZSCH, Germany) was performed from room temperature to 800 °C at a heating rate of 10 °C min^−1^ and a N_2_ flow rate of 20 mL min^−1^. Gas chromatography (GC, Agilent 7890A) equipped with a capillary column (HP-5, 30 m × 0.25 mm) using a flame ionization detector. ^1^H and ^13^C nuclear magnetic resonance (NMR) spectra were recorded by a Bruker Advance III 400 MHz NMR spectrometer (Bruker BioSpin Corporation, Fällanden, Switzerland). Inductively coupled plasma optical emission spectroscopy (ICP-OES) was conducted by ICP-OES 7300DV (PerkinElmer). The sample was firstly calcinated at 1000 °C in the air for 12 h to burn out organic moieties. The residue was dissolved by aqua regia and then diluted by water for ICP-OES testing. X-ray photoelectron spectroscopy (XPS) was recorded by ESCALAB 250Xi equipped with Al Kα radiation (1486.6 eV, 200 W) on sample powder pressed pellet.

## 3. Results and discussion

### 3.1. Characterization of COFs

Efforts to produce amine-linked COF using this new strategy commenced with the layered TFPPy–PyTTA–COF that was synthesized through the condensation of TFPPy and PyTTA under solvothermal condition. The post-synthetic modification of TFPPy–PyTTA–COF has been achieved through three main steps: (1) direct reduction of imine linkages to give corresponding amine-linked A-TFPPy–PyTTA–COF; (2) grafting alkyl sulfonic acid groups on the channel walls through aminolysis reaction with 1,3-propane sultone;^32^ (3) ionization of SO_3_H@TFPPy–PyTTA–COF to Co^2+^@TFPPy–PyTTA–COF through metathetical reaction of alkyl sulfonic acid with cobalt acetate. General pathway is represented schematically in Scheme 1.

**Fig. 1:**
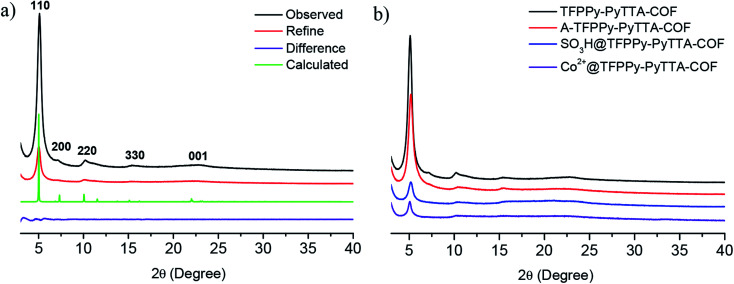
(a) PXRD patterns of TFPPy–PyTTA–COF. Observed XRD pattern (black) and profiles simulated using the Pawley refinement (red), their difference (violet), and AA-stacking modes (green) of the TFPPy–PyTTA–COF; (b) comparison of PXRD patterns of TFPPy–PyTTA–COF, A-TFPPy–PyTTA–COF, SO_3_H@TFPPy–PyTTA–COF and Co^2+^@TFPPy–PyTTA–COF.

**Fig. 2:**
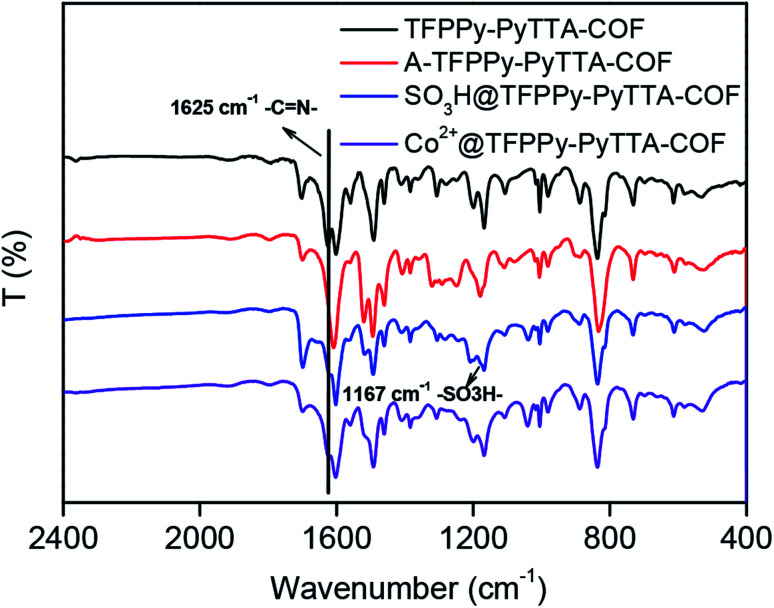
FT-IR spectra of TFPPy–PyTTA–COF, A-TFPPy–PyTTA–COF, SO_3_H@TFPPy–PyTTA–COF and Co^2+^@TFPPy–PyTTA–COF.

The crystalline structure and unit cell parameter of TFPPy–PyTTA–COF were analyzed by powder X-ray diffraction (PXRD) in combination with structural simulations. Diffraction peaks of TFPPy–PyTTA–COF appeared at 5.1°, 7.1°, 10.2°, 15.2° and 22.5° which were attributed to the (110), (200), (220), (330) and (001) facets, respectively (Fig. 1a, black curve). The use of lattice modeling and Pawley refinement processes give an eclipsed AA stacking model that could reproduce the PXRD pattern in the peak position and intensity well (Fig. 1a, red curve). The Pawley refinement (Fig. 1a, blue curve) using the unit cell parameters of *a* = 24.96 Å, *b* = 23.90 Å, *c* = 3.89 Å and *α* = *β* = *γ* = 90°, confirmed the peak assignment, as evidenced by their negligible difference (Fig. 1a, violet curve). Evidently, the crystalline structure of TFPPy–PyTTA–COF was in accordance with that reported previously.^28^ After the reduction of imine linkages, A-TFPPy–PyTTA–COF exhibited an XRD pattern similar to that of TFPPy–PyTTA–COF, indicating that it possesses a similar crystal structure. Grafting alkyl sulfonic acid and subsequent ionization did not change the position of diffraction peaks, revealing that the pristine crystal structure of TFPPy–PyTTA–COF was remained after three-step modification (Fig. 1b). The peak intensity of the (100) facet was decreased which was attributed to the occupation of the pores by the grafted functional groups. Besides, unpleasant damage to the crystallization of COFs may happen during the post-modification processes.^24^ Successful reduction of the imine linkages of TFPPy–PyTTA–COF was confirmed by FTIR spectroscopy (Fig. 2), where the characteristic stretching peak of imine group at 1627 cm^−1^ disappeared. SO_3_H@TFPPy–PyTTA–COF exhibited the emergence of a characteristic peak of –SO_3_H (1167 cm^−1^), indicating the successful grafting of alkyl sulfonic acid groups on the channel walls. The introduction of Co^2+^ into the COF skeletons was also confirmed by ICP-OES technology. The Co^2+^ content was measured to be 3.6 wt% (7.0 wt% in theory), which means that about 51% imine linkages were successfully modified.

**Fig. 3:**
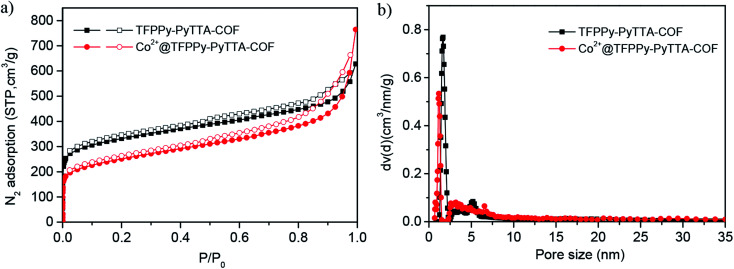
(a) N_2_ adsorption–desorption isotherms recorded at 77 K and (b) the corresponding DFT pore-size distributions of TFPPy–PyTTA–COF and Co^2+^@TFPPy–PyTTA–COF.

**Fig. 4:**
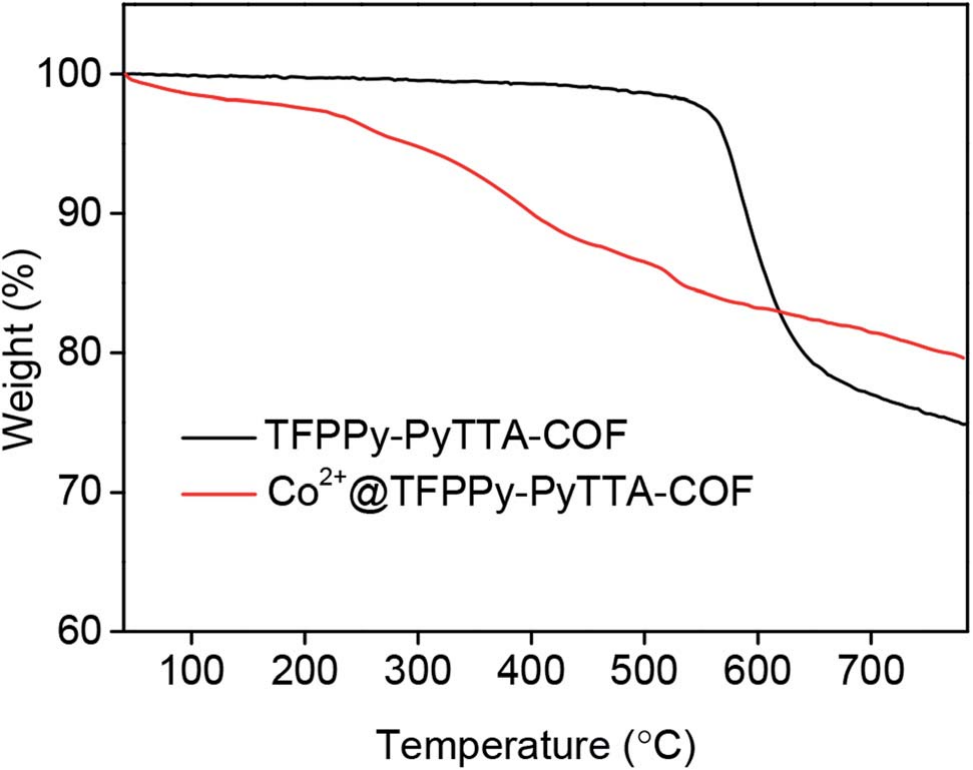
TGA curves of TFPPy–PyTTA–COF and Co^2+^@TFPPy–PyTTA–COF.

**Fig. 5:**
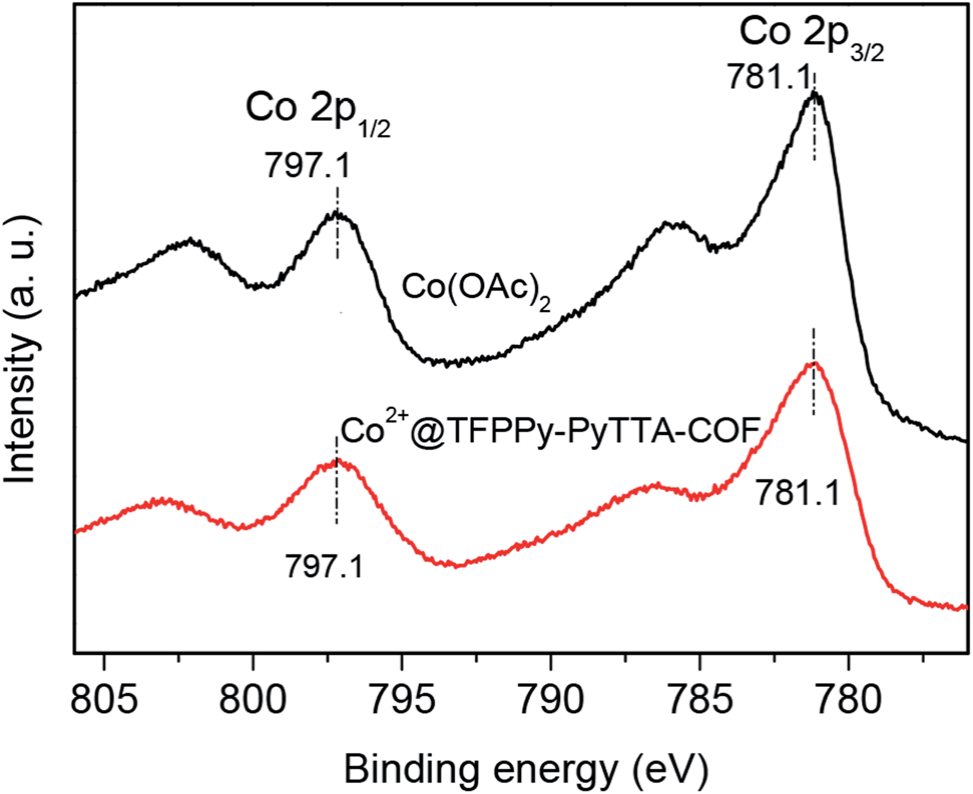
XPS spectra of Co(OAc)_2_ and Co^2+^@TFPPy–PyTTA–COF.

The porous properties of the COFs were characterized by nitrogen adsorption–desorption isotherms measured at 77 K. As shown in Fig. 3a, both pristine TFPPy–PyTTA–COF and modified Co^2+^@TFPPy–PyTTA–COF exhibited the typical type-IV adsorption isotherms, indicative of mesoporous characteristics. Their BET surface areas were measured to be 1120, and 600 m^2^ g^−1^, while their corresponding total pore volumes were calculated to be 1.14, and 0.84 cm^3^ g^−1^, respectively. The pore size was decreased from 1.7 to 1.2 nm (Fig. 3b), which is consistent with the fact that the pores were occupied by the grafted functional groups. From the PXRD and adsorption isotherm results, it can be deduced that the ordered framework structure was remained to a certain extent even if the TFPPy–PyTTA–COF underwent three modifications. The thermal behavior of these two COFs was detected by thermogravimetric analysis (TGA). The decomposition temperature was 550 and 230 °C for TFPPy–PyTTA–COF and Co^2+^@TFPPy–PyTTA–COF, respectively (Fig. 4). Although the thermal stability of the material was decreased after modifications, it is still practicable for Co^2+^@TFPPy–PyTTA–COF used as catalysts for the cycloaddition of epoxides and CO_2_. X-ray photoelectron spectroscopy (XPS) was further performed to determine the valence state of loaded Co^2+^ (Fig. 5). In comparison with Co(OAc)_2_·4H_2_O, both the Co 2p_1/2_ and Co 2p_3/2_ signals for Co^2+^@TFPPy–PyTTA–COF showed the same energy values, indicating the free state of Co^2+^ in the frameworks.

### 3.2. CO_2_ adsorption of COFs

**Fig. 6:**
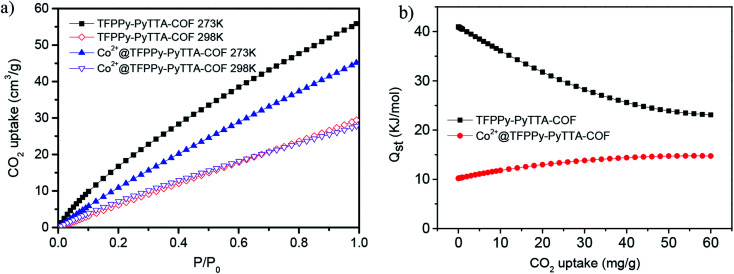
(a) CO_2_ adsorption isotherms of COFs under 273 K (solid symbol) and 298 K (open symbol) and (b) their respective isosteric heats of adsorption for CO_2_ of TFPPy–PyTTA–COF and Co^2+^@TFPPy–PyTTA–COF.

The CO_2_ adsorption capability of TFPPy–PyTTA–COF and Co^2+^@TFPPy–PyTTA–COF was investigated in this research. The CO_2_ adsorption isotherms of both COFs were measured at pressures up to 1 bar at 273 K and 298 K, respectively. TFPPy–PyTTA–COF and Co^2+^@TFPPy–PyTTA–COF showed CO_2_ uptake capacities of 56.1, and 45.4 cm^3^ g^−1^ (*ca.* 11.0 wt% and 8.9 wt%) at 273 K and 1 bar, which decreased to 29.5 and 27.7 cm^3^ g^−1^ (*ca.* 5.8 wt% and 5.4 wt%), respectively, when the temperature was increased to 298 K, 1 bar (Fig. 6a). The CO_2_ adsorption capacity of TFPPy–PyTTA–COF was decreased after modifications at 273 K, which can be ascribed to the drastic decrease in the surface area. Although a decreased pore size and introduction of highly charged groups on the channel walls can both enhance the interaction between CO_2_ molecules and the pore surface of the COF,^33^ the CO_2_ adsorption capacity seems to be dominated by the decreased BET surface area in this work. The CO_2_ adsorption enthalpies were calculated for TFPPy–PyTTA–COF and Co^2+^@TFPPy–PyTTA–COF (Fig. 6b), in accordance with the result of their CO_2_ adsorption ability. High CO_2_ adsorption capability of the COFs paves the way for Co^2+^@TFPPy–PyTTA–COF to exhibit excellent catalytic performance towards the cycloaddition of CO_2_ to epoxides.

### 3.3. Catalytic performance of COFs

To evaluate the catalytic performance of the resulting Co^2+^@TFPPy–PyTTA–COF, the cycloaddition of CO_2_ to epoxides was chosen as the probe reaction. The reactions were performed at 100 °C, 3.0 MPa CO_2_ and reaction time was 48 h. The experimental results are shown in Table 1. In the absence of the catalyst, a conversion of 38% of epichlorohydrin to the cyclic carbonate was obtained under the above reaction conditions (Table 1, entry 1), while a conversion as high as 99% (98% isolated yield) was observed in the presence of Co^2+^@TFPPy–PyTTA–COF (Table 1, entry 2), which is identical to that of Co(OAc)_2_·4H_2_O (99% conversion, Table 1, entry 3), suggesting that the heterogeneous Co^2+^@TFPPy–PyTTA–COF possessed an excellent catalytic performance that can only be achieved by the corresponding homogenous counterpart. The catalytic activity of the Co^2+^@TFPPy–PyTTA–COF in cycloaddition of CO_2_ to different epoxides under the identical conditions has been examined. The epoxides used here are propylene oxide, 1,2-epoxyhexane, butyl glycidyl ether, styrene oxide, and 1,2-epoxyoctane (Table 1, entries 3–7). The conversion for these reactions was 99%, 98%, 96%, 99%, and 70% and the isolated yield was 99%, 96%, 96%, 97%, and 65%, respectively. These results exhibited that Co^2+^@TFPPy–PyTTA–COF possesses excellent catalytic activity towards the cycloaddition of epoxides and CO_2_. This reaction could follow a Lewis acid catalysis mechanism where Co^2+^ and sulfonate act as Lewis acid and nucleophile (co-catalyst), respectively.^34^

**Table 1:** Cycloaddition reactions of CO_2_ and various epoxides over Co^2+^@TFPPy–PyTTA–COF catalysts^a^

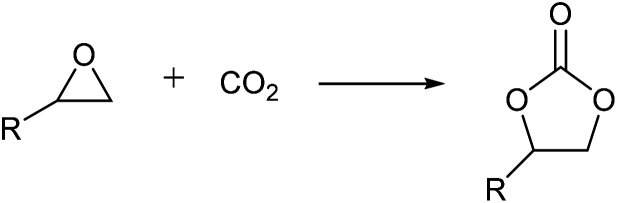				
Entry	Catalyst	Substrate	Conversion^b^ (%)	Yield^c^ (%)
1	—	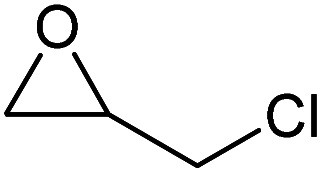	38	36
2	Co^2+^@TFPPy–PyTTA–COF	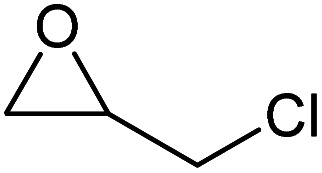	>99	98
3	Co(OAc)_2_·4H_2_O	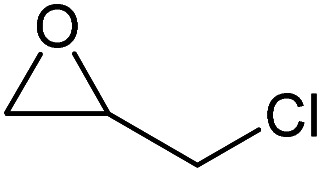	>99	97
4	Co^2+^@TFPPy–PyTTA–COF	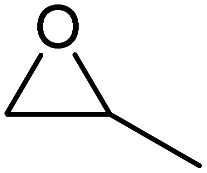	>99	99
5	Co^2+^@TFPPy–PyTTA–COF	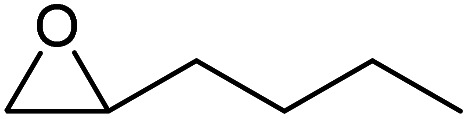	98	96
6	Co^2+^@TFPPy–PyTTA–COF	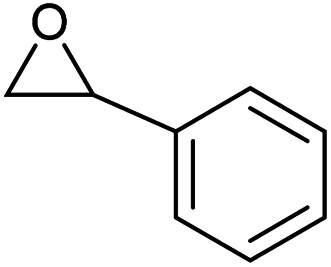	96	96
7	Co^2+^@TFPPy–PyTTA–COF	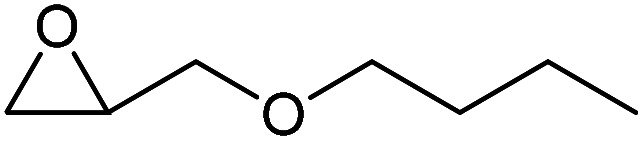	99	97
8	Co^2+^@TFPPy–PyTTA–COF	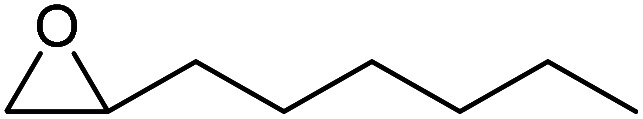	70	65

a Reaction conditions: epoxides (7.5 mmol), Co^2+^@TFPPy–PyTTA–COF (24.4 mg), 100 °C, 48 h, *P*(CO_2_) = 3.0 MPa.

b Conversion was determined by Gas Chromatography (GC).

c Isolated yield.

**Fig. 7:**
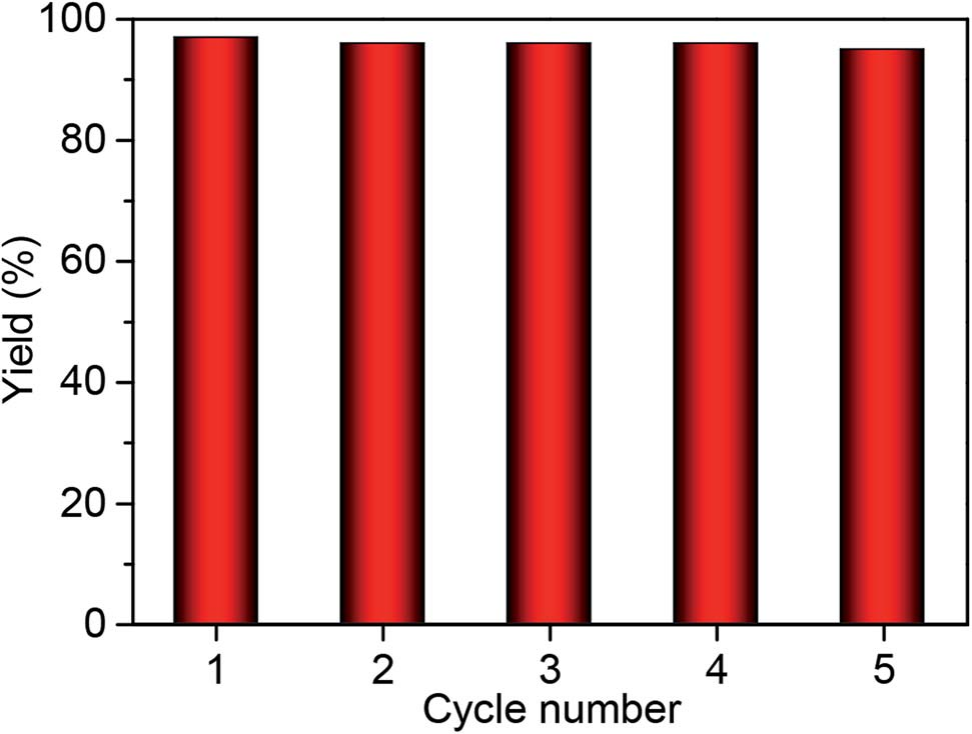
Catalytic activity of recycled Co^2+^@TFPPy–PyTTA–COF.

**Fig. 8:**
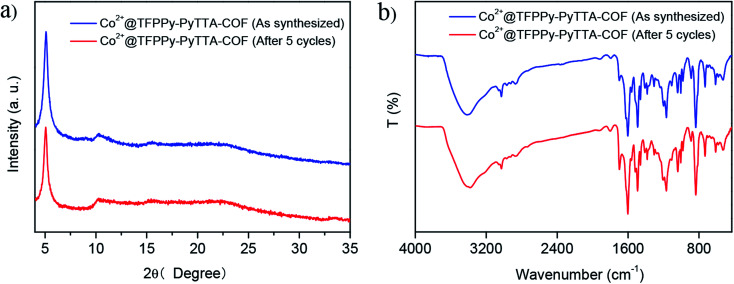
XRD patterns (a) and FT-IR spectra (b) of Co^2+^@TFPPy–PyTTA–COF before and after 5 cycles.

The heterogeneity of Co^2+^@TFPPy–PyTTA–COF was confirmed by hot filtration of the catalyst after 2 h of reaction, which led to negligible addition in the product yield up to 24 h after filtration. This result revealed that Co^2+^@TFPPy–PyTTA–COF was a heterogeneous catalyst, and no catalytically active species were released into solution. The reusability of the as-synthesized Co^2+^@TFPPy–PyTTA–COF and reproducibility of catalytic performance have been evaluated based on experimental results of constant cyclic tests. In each cycle, Co^2+^@TFPPy–PyTTA–COF was removed by centrifugation and then rinsed with epichlorohydrin. After drying, the catalyst was reused for the next run. The yields of cyclic carbonate in the first five consecutive runs are given in Fig. 7. It was indicated that the Co^2+^@TFPPy–PyTTA–COF can be reused at least five times without significant loss of activity in the fifth run. After five runs, the PXRD pattern of Co^2+^@TFPPy–PyTTA–COF was well retained (Fig. 8a) and FTIR spectra showed no obvious changes (Fig. 8b), indicating that Co^2+^@TFPPy–PyTTA–COF can be considered as a renewable and stable catalyst.

## 4. Conclusions

In summary, we have developed a new post-synthetic modification strategy that introduced functional groups on the linkages of a layer COF material. The imine linkages of the pristine TFPPy–PyTTA–COF were first reduced into amine-linkages and further modification was carried out based on the amine-linkages that underwent aminolysis reaction with 1,3-propane sultone and subsequent metathetical reaction with cobalt acetate [Co(OAc)_2_], generating an ionic COF with uniformly distributed catalytic sites on the channel walls of the COF. The catalytic performance of the resulting COF was evaluated by the cycloaddition of epoxides and CO_2_. Our experiments showed that high catalytic activity and good recycle ability were observed for the COF catalyst. This strategy opens a way to modify postsynthetically COF materials for multifunctional exploration.

## Conflicts of interest

There are no conflicts to declare.

## Supplementary Material
